# Comment on Alghnam et al. The Association between Obesity and Chronic Conditions: Results from a Large Electronic Health Records System in Saudi Arabia. *Int. J. Environ. Res. Public Health* 2021, *18*, 12361

**DOI:** 10.3390/ijerph19169846

**Published:** 2022-08-10

**Authors:** Rakan M. Alsarwani

**Affiliations:** College of Medicine, Umm Al-Qura University, Makkah 24382, Saudi Arabia; rakansi@outlook.sa

I have read the interesting and informative paper recently published by Alghnam and colleagues that examined associations between obesity and diabetes (DM) and hypertension (HTN) [1]. This cross-sectional study involved a population-based sample across several Saudi Arabian regions, providing a clearer focus of the burden of these issues in the country. There are, however, several points that I believe require clarification. In the first instance, the authors identified diabetic and hypertensive patients in large part on the basis of their prescribed medications. However, this may have resulted in the inclusion of some patients without those conditions but who had been prescribed the medication in question, as some of the prescribed medications can also be used in other clinical conditions, and their use is not solely limited to DM or HTN. For example, metformin may be prescribed for female patients with polycystic ovary syndrome [2], ACE inhibitors can be used in acute coronary syndrome [3], and β-blockers and CCB can also be used to treat certain cardiac arrhythmias (e.g., atrial fibrillation) [4]. Given this, some such patients may have been misidentified as diabetic or hypertensive.

Secondly, patients with type 1 diabetes (T1DM) who depend on insulin from the onset of the disease were less likely to have been included in the study; this is because insulin, despite its near-exclusive indication for DM, was not considered when identifying diabetic patients. The system captures the discharge diagnosis only, and hence, such patients who have T1DM as a secondary diagnosis would not have been identified. Furthermore, insulin can be used to augment or even replace oral glycemic medications in patients with type 2 diabetes mellitus, which, if not addressed, may have resulted in those patients also being overlooked.

Thirdly, cancer was among the diagnoses extracted from the system; however, it is unclear why it was included as it was neither discussed in the results section nor represented in the tables/graphs.

Finally, the investigators classified patients into diabetic and non-diabetic groups, and hypertensive and non-hypertensive groups, but did not account for patients who may have had both conditions. While the study reported a significant association between BMI and HTN (*p* < 0.01), the potentially confounding influence of DM was not controlled for. Similarly, a significant association between BMI and DM (*p* < 0.01) was found, without controlling for the potentially confounding influence of HTN. Hence, it is conceivable that the significant associations found between BMI and HTN and between BMI and DM may be due, at least in part, to patients with both HTN and DM as the “diabetic/non-diabetic” groups did not filter out patients with HTN and DM was not excluded in the “hypertensive/non-hypertensive” groups.

While the study provides important information regarding the prevalence of obesity and its association with DM and HTN, I encourage the authors to further clarify the points raised in this letter, as such clarification would be a valuable addition to the existing literature.
